# Domestic

**Published:** 1840-07-11

**Authors:** 


### DOMESTIC.

*Quarterly Report of the Obstetric Practice of the Philadelphia Dispensary for Fourth, Fifth, and Sixth months*, 1840. By Joseph Warrington, M. D., Accoucheur.

Twenty-six women have been delivered at full time, and one other attended on account of abortion at the fourth month of gestation.

Of the children born at full time, sixteen were girls, and ten boys.

The average duration of labour in twenty-two cases was nine hours,—the extremes being one and fifty hours.

The average time required for the spontaneous delivery of the placenta in twenty-four cases, was about twenty-six minutes,—the extremes being five and one hundred and ninety-five minutes.

In twenty cases of labour in which the presentations of the fœtus were carefully noted, eleven were in the first, seven in the second, one in the fourth position of the vertex, and one in the second position of the breech.

Abortion was brought on in the case referred to by external violence.

All the women have recovered without accident excepting one case, in which prolapsus uteri was brought on by rising and making exertion on the second day after delivery.

Of the children, three were still-born,—one apparently in consequence of excessive hæmorrhage through the placenta situated at the os uteri,—another appeared to have died in utero some weeks before delivery,—and one died asphyxied, after the use of ergot to terminate a labour which had continued fifty hours.

The rest of the children have done well.

The forceps were successfully used to deliver in one case in which labour had continued forty hours,—the child's head being strongly arrested in the pelvis in the fourth position.

